# Influence of lipid metabolism disorders on venous thrombosis risk

**DOI:** 10.5937/jomb0-27106

**Published:** 2021-06-05

**Authors:** Igor Spasić, Milan Ubavić, Zorica Šumarac, Maša Todorović, Biljana Vučković

**Affiliations:** 1 University of Novi Sad, Faculty of Medicine, Department of Pathophysiology, Novi Sad; 2 Institute of Laboratory Diagnostics Medlab, Novi Sad; 3 University Business Academy, Faculty of Pharmacy Novi Sad, Novi Sad; 4 Clinical Center of Serbia, Belgrade

**Keywords:** DVT, hemostasis, hyperlioproteinemia, lipidmetabolism, Lp (a) hyperlipoproteinemia, DVT, hemostaza, hiperlipoptroteinemija, metabolizam lipida, Lp(a) hiperlipoproteinemija

## Abstract

**Background: **To investigate the influence of lipid metabolism disorders on the risk of deep vein thrombosis.

**Methods:** A total of 200 subjects participated in the study, 100 of whom experienced DVT with or without PTE, and 100 healthy subjects representing the control group. We classified patients and controls in terms of serum concentrations of chylomicrons, LDL, IDL, VLDL, and HDL particles, as those with or without hyperlipoproteinemia and in terms of serum Lp (a) lipoprotein levels, as those with hyperLp (a) lipoproteinemia (serum Lp (a) values >0.3 g/L) and those without hyperLp (a) lipoproteinemia (serum Lp (a) values <0.3 g/L). Based on the modified and supplemented Fredrickson classification, participants with verified existences of hyperlipoproteinemia were classified into subgroups based on the type of hyperlipoproteinemia. Unconditional logistic regression was used to calculate ORs with 95% CIS as a measure of the relative risks for venous thrombosis in participants with hyperlipoproteinemia compared with those without hyperlipoproteinemia. The analysis was adjusted for all potential confounders (age, sex, obesity) related to the functionality of the lipid metabolism, and at the same time, may have an impact on the risk of venous thrombosis.

**Results:** The results of the comparison of the mean values of individual lipid status parameters between the patient group and the control group clearly indicate higher concentrations of total cholesterol (5.93 mmol/L vs. 5.52 mmol/L), total triglycerides (1.58 mmol/L vs. 1.50 mmol/L), and LDL-cholesterol (3.83 mmol/L vs. 3.44 mmol/L) in the patient group relative to the control group, with a statistically significant difference observed only in the case of LDL-cholesterol concentrations. We have found that type IIa hyperlipoproteinemia is associated with a nearly double increased risk for deep vein thrombosis (OR 1.99; Cl 1.01-3.90), while type IIb, IV, or hyperLp (a) lipoproteinemia did not influence the risk (OR 1.22; 95% Cl 0.79-1.84; OR 0.89; 95% Cl 0.52-1.54 OR 1.85; 95% CI 0.84-4.04).

**Conclusions:** Hypercholesterolemia doubles the risk of deep vein thrombosis development.

## Abstract (sr)

Uvod: Cilj je bio da se istraži uticaj poremećaja metabolizma lipida na rizik od razvoja tromboze dubokih vena.

**Metode:** U istraživanju je učestvovalo ukupno 200 ispitanika, od kojih je 100 doživelo DVT sa ili bez PTE i 100 zdravih ispitanika koji su predstavljali kontrolnu grupu. Pacijente i kontrolnu grupu klasifikovali smo prema serumskim koncentracijama hilomikrona, LDL, IDL, VLDL i HDL čestica, na one sa ili bez hiperlipoproteinemije i prema nivou Lp (a) lipoproteina u serumu, na one sa hiperLp (a) lipoproteinemijom (serumske vrednosti Lp (a) > 0,3 g/L) i one bez hiperLp (a) lipoproteinemije (vrednosti Lp (a) u serumu < 0,3 g/L). Na osnovu modifikovane i dopunjene Fredriksonove klasifikacije ispitanici sa dokazanim postojanjem hiperlipoproteinemije razvrstani su u podgrupe prema tipu hiperlipoproteinemije. Ne kondicionalna logistička regresija je korištena za izračunavanje OR-a sa 95% CI kao mere relativnog rizika od venske tromboze kod ispitanika sa hiperlipoproteinemijom u poređenju sa onima bez hiperlipoproteinemije. Analiza je prilagođena za sve potencijalne "confoundere" (uzrast, pol, gojaznost) koji se odnose na funkcionalnost metabolizma lipida, a istovremeno mogu imati uticaj na rizik od nastanka venske tromboze.

**Rezultati:** Rezultati poređenja srednjih vrednosti pojedinačnih parametara lipidnog statusa između grupe pacijenata i kontrolne grupe jasno ukazuju na veće koncentracije ukupnog holesterola (5,93 mmol/L naspram 5,52 mmol/L), ukupnih triglicerida (1,58 mmol/L naspram 1,50 mmol/L) i LDL-holesterola (3,83 mmol/L naspram 3,44 mmol/L) u grupi pacijenata u odnosu na kontrolnu grupu, sa statistički značajnom razlikom uočenom samo u slučaju koncentracije LDL-holesterola. Otkriveno je da je hiperlipoproteinemija tipa IIa povezana sa gotovo dvostruko većim rizikom od duboke venske tromboze (OR 1,99; Cl 1,01-3, 90), dok lipoproteinemija tipa IIb, IV ili hyperLp (a) nije uticala na rizik (OR 1,22; 95% Cl 0,79-1,84; OR 0,89; 95% Cl 0,52-1,54 ILI 1,85; 95% CI 0,84-4,04).

Zaključak: Hiperholesterolemija udvostručuje rizik od razvoja tromboze dubokih vena.

## Introduction

Thrombosis represents a life-long intravascular or intracardiac formation of blood clots as a result of a disorder of the hemostatic mechanism, that is, its constituent factors such as platelets, coagulation factors, fibrinolysis factors, and inhibitors of coagulation and fibrinolysis.

The incidence of venous thrombosis in Europe is 160-180/100,000 [1]. The clinical significance of venous thrombosis is reflected in high mortality rate [2], high recurrence rate [3], and complications that may occur after the first episode of venous thrombosis, described as a set of symptoms and signs collectively referred to as Postthrombotic Syndrome (PTS).

The underlying pathophysiological mechanism of venous thrombosis is explained by Virchow's Triassic, which implies the existence of slow blood flow and the termination of its laminar movement, a change in the vessel wall, and changes in blood composition [4] [5]. The risk factors for DVT occurrence are roughly classified into those acquired and those that have been inherited. Some of the accepted acquired factors include age, immobilization, pregnancy and puerperium, malignancy, antiphospholipid antibodies, oral contraceptives, and hormone therapy, as well as life habits. As far as the inherited factors are concerned, these include factor V Leiden mutation, factor II (prothrombin) G20210A mutation, deficiency of natural coagulation inhibitors (antithrombin, protein C, protein S), and non-0 blood type [6] [7] [8]. It is essential to emphasize the influence of the multiplication of risk factors, which basically shows that the combined effect of two or more risk factors multiplies their detrimental impact. For years, blood stasis was the most researched primary pathophysiological mechanism of vein thrombosis. More recent studies have made an effort to find a connection between the risk factors and pathophysiological mechanisms of arterial and venous thrombosis, based on the concept of endothelial dysfunction. Common risk factors for arterial and venous thrombosis include age, immobi-lization, obesity, metabolic syndrome and diabetes, smoking, hypertension, malignancy, infection, and lipid metabolism disorders [9] [10] [11] [12]. Defects in cholesterol metabolism and hypercholesterolemia, which are major risk factors for atherosclerosis, have been shown to affect venous thrombosis risk, as well. Still, there is inconsistency in the results of different studies.

Additionally, it is known that one of the most atherogenic lipid particles, which is recognized as a significant risk factor for arterial thrombosis development, is Lp (a) lipoprotein [13] [14] [15]. The structure of Lp (a) lipoprotein particle is similar to that of low-density lipoprotein (LDL) in terms of size and lipid composition, as well as the presence of apolipoprotein B100 (apo B100). The similarity of apo (a) with plasminogen was observed in the form of domains of inactive serine protease, whose amino acid sequence was 94% coincident with the amino acid sequence of plasminogen. Replacing serine with arginine at the activation site of this sequence results in the activation of the protease in the same way it does in the case of plasminogen, indicating the possibility of apo (a) activation by the presence of a tissue plasminogen activator (t-PA), urokinase and streptokinase. By finding a similarity between the structure of plasminogen and the Lp (a) particle, it was assumed that there was a connection between atherogenesis and thrombogenesis as well, with Lp (a) as the connecting link [16]. A clear pathophysiological mechanism has not been proven, but the premise is that Lp (a) lipoprotein intervenes with the fibrinolysis system and competes with plasminogen for binding sites on endothelial cells, thereby inhibiting fibrinolysis and promoting intravascular coagulation [17]. Studies evaluating the role of this specific lipoprotein in venous thrombosis appearance are scarce and inconsistent, as well.

Venous thrombosis is classified as primary (spontaneous) and provoked. This classification is very important because of the different therapeutic approaches between them. The long-term use of anticoagulant therapy in patients with spontaneous deep vein thrombosis has been indicated with the aim of reducing the high frequency (15–25%) of symptomatic propagation of the thrombosis process and prevention of recurrence of venous thrombosis [18]. However, bleeding remains the most common side effect of anticoagulant therapy. Lifelong oral anticoagulant therapy carries the risk of major hemorrhage of 2–3% per year [19]. Thus, the primary goal is to define and detect all pathophysiological mechanisms and risk factors, which will ultimately lead to a reduction in the number of patients on lifelong OAT.

That is why the focus of newer researches, including ours, is to investigate the effect of lipid metabolism and its disorders as a contributing factor in the complex pathophysiology of venous thromboembolism.

## Materials and Methods

### Study design

The research was conducted within the Center for Laboratory Medicine, Clinical Center of Vojvodina and Department of Pathophysiology and Laboratory Medicine, Faculty of Medicine in Novi Sad, from October 2018 to July 2019. A total of 200 subjects participated in the study, 100 of whom experienced DVT with or without PTE, and 100 healthy subjects representing the control group. Data were obtained from patient records and supporting medical records.

The patient group consisted of 48 men (48%) and 52 women (52%). The youngest respondent was 19 years old, and the eldest 88, while the average age of the respondents was 52 years. The age range for inclusion in the study was 18 years and older. The control group of healthy subjects consisted of 100 persons, out of which 51 (51%) were male, and 49 (49%) were female. The average age was 50 years. The youngest respondent was 19, and the oldest was 87.

### Inclusion/exclusion criteria

The basic criteria for the inclusion of the study participants were that they had experienced DVT or PTE. The diagnosis of DVT was confirmed based on clinical history and examination and supplementary imaging diagnostics in the form of a Duplex scan. The diagnosis of PTE was confirmed by lung perfusion scintigraphy with angiography. In each case, it was necessary that at least 3 months had elapsed since the clinical event, which excluded the possible impact of the acute phase response on the values of the parameters tested. Additionally, patients were enrolled in the study at least 3 months after discontinuing oral anticoagulant therapy in order to avoid the influence of these drugs on the functionality of the hemostatic mechanism.

**Figure 1:**
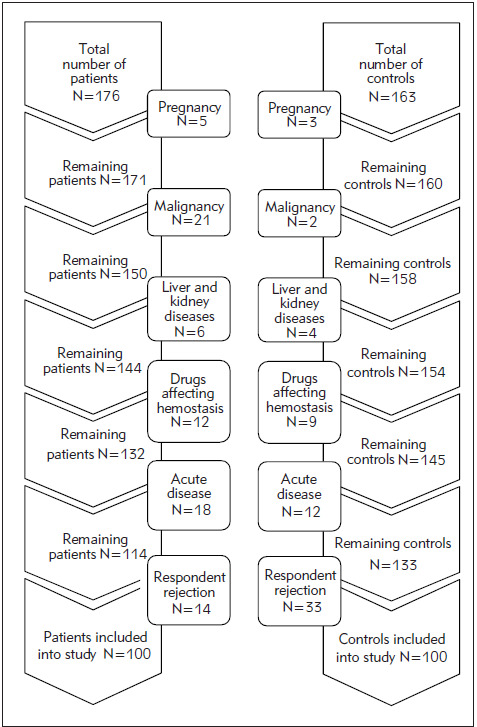
Selection of subjects in the patient and control group

The exclusion criteria of the study included previously diagnosed disorders of hemostatic mechanism, current therapy that can affect the functionality of hemostatic mechanism with the exception of antiplatelet drugs, acute illness 6 weeks before blood sampling or at the time of sampling, malignancy, pregnancy, and puerperium, severe mental illness, liver and kidney diseases, autoimmune diseases, refusal of the subject to sign informed consent. The exclusion criteria for the control group of subjects were identical to the criteria for the patient group (Figure 1).

We classified patients and controls in terms of serum concentrations of chylomicrons, LDL, intermediate-density lipoprotein (IDL), very low-density lipoprotein (VLDL), and high-density lipoprotein (HDL) particles, into two categories - those with or without hyperlipoproteinemia. Additionally, in terms of serum Lp (a) lipoprotein levels, subjects were classified into those with hyperLp (a) lipoproteinemia (serum Lp (a) values >0.3 g/L) and those without hyper Lp (a) lipoproteinemia (serum Lp (a) values <0.3 g/L) within both the patient and the control group.

Finally, parameters used for diagnosing the type of hyperlipoproteinemia (serum levels of total cholesterol and triglycerides, HDL-cholesterol, refrigerated test) were based on the modified and supplemented Fredrickson classification participants with verified existences of hyperlipoproteinemia and classified into subgroups based on the type of hyperlipoproteinemia.

### Definition and data collection

The analysis of blood lipids and Lp (a): serum extracted from whole blood after coagulation and a two-hour incubation period of the sample at room temperature were used for blood lipids and Lp (a) lipoprotein analyses. Lipid status parameters were assayed in fresh serum samples, while Lp (a) lipoproteins were determined from serum that was frozen at -20°C for less than a month.

Serum cholesterol concentrations as well as serum triglyceride concentrations were determined by a standardized enzyme procedure. The determination of cholesterol in the HDL fraction was performed by a direct enzymatic method for the quantitative determination of HDL cholesterol in human serum. LDL cholesterol values (Friedewald et al.) and non-HDL cholesterol (total cholesterol-HDL cholesterol), as well as atherosclerosis index (LDL cholesterol/HDL cholesterol) and atherogenic ratios (total cholesterol/HDL cholesterol and non-HDL cholesterol/HDL cholesterol), were obtained by computation. Additionally, the characteristics of the serum after an 18-24-hour period at a temperature of +4°C was also evaluated.

Serum Lp (a) lipoprotein concentration was determined by the immunoturbidimetric method of Latex agglutination, using latex particles with monoclonal antibodies highly specific for Lp (a) lipoprotein, automatically. The Latex reagent contains microparticles of equal size, coated with monoclonal antibodies highly specific for Lp (a) lipoprotein. After mixing the serum sample with the Latex reagent, agglutination, which is directly proportional to the concentration of Lp (a) lipoprotein in the sample, occurs and is determined by a kinetic assay, measuring the increased absorption at 570 nm caused by the presence of the aggregate. The results are expressed in g/L.

### Statistical analysis

Statistical data processing was performed using SPSS software, version 23.0 (SPSS, Chicago, IL, USA). Descriptive statistic methods were used to show the basic characteristics of the respondents. The Mann-Whitney U test was used to test the statistical significance of differences for continuous variables having a normal distribution. Pearson's c^2^ test was used to evaluate the differences in the frequency of individual parameters between different groups of subjects. A P-value of less than 0.05 was considered statistically significant in each of the before mentioned tests. Unconditional logistic regression was used to calculate ORs with 95% CIs as a measure of the relative risks for venous thrombosis in participants with hyperlipoproteinemia compared with those without it. Lastly, the analysis was adjusted for all potential confounders (age, sex, obesity) related to the functionality of the lipid metabolism, which may have an impact on the risk of venous thrombosis.

## Results

The basic characteristics of the respondents included in the study are presented in Table 1. Recognized risk factors for the development of venous thrombosis are, as expected, more frequently present in patients than in the control group. An analysis of the prevalence of classical arterial thrombosis risk factors reveals that patients who have experienced venous thrombosis are more likely to be obese (22% vs. 16%), smokers (31% vs. 24%), or have hypertension (41% vs. 29%) compared to healthy controls. Hyperlipoproteinemia (69% vs. 54%) and hyperLp (a) lipoproteinemia (20% vs. 12%) were more commonly reported in patients with venous thrombosis than in the healthy controls.

**Table 1:** Clinical characteristics of the subjects^1^ ^†^Classic risk factors include surgery, malignancy, immobility, trauma, gypsum immobilization, use of hormonal medicaments and longer travel, ^1^Values are n(%) unless otherwise indicated.

	Patients (n=100)	Controls (n=100)
General characteristics		
Male	48 (48)	51 (51)
Female	52 (52)	49 (49)
Age (years)	52 (19-88)	50 (19-87)
Body mass index, kg/m^2^	27 (17-39)	26 (18-37)
Classic risk factors for venous thrombosis		
Present^† ^	44 (44)	15 (15)
Absent^†^	56 (56)	85 (85)
Classic risk factors for arterial thrombosis		
Obesity	22 (22)	16 (16)
Smoking	31 (31)	24 (24)
Hypertension	41 (41)	29 (29)
Prevalence of lipid metabolism disorders		
Hyperlipoproteinemia	69 (69)	54 (54)
Hyper Lp (a) Lipoproteinemia	20 (20)	12 (12)

**Figure 2:**
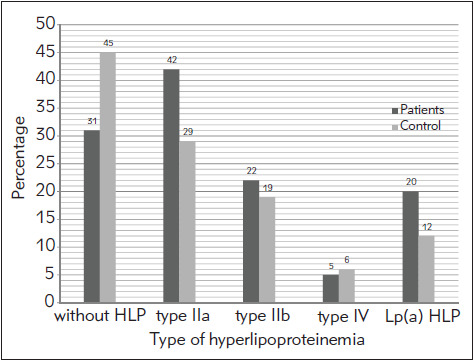
Distribution of hyperlipoproteinemia type in the patient group and the control group of subjects

By analyzing the distribution of hyperlipoproteinemia types in the patient group and the control group, results showing a more frequent presence of type IIa and IIb hyperlipoproteinemia in the patient group compared to the control group were obtained, as shown in Figure 2.

The results of the comparison of the mean values of individual lipid status parameters between the patient group and the control group clearly indicate higher concentrations of total cholesterol (5.93 mmol/L vs. 5.52 mmol/L), total triglycerides (1.58 mmol/L vs. 1.50 mmol/L), and LDL-cholesterol (3.83 mmol/L vs. 3.44 mmol/L) in the patient group than in the control group, with a statistically significant difference observed only in the case of LDL-cholesterol concentrations, as shown in Table 2.

**Table 2:** Comparison of mean values of individual lipid status parameters between the patient group and the control group of healthy subjects Values are given as means (range); LDL-low density lipo-proteins; HDL-high density lipoproteins

Lipid status parameters (mmol/l)	Patients (n=100)	Controls (n=100)	p
Total cholesterol	5,93 (3.32-9.20)	5,52 (2.78-8.09)	0,12
Total triglycerides	1,58 (0.24-6.70)	1,50 (0.41-4.32)	0,550
LDL-cholesterol	3,83 (1.49-6.26)	3,44 (1.29-5.56)	0,005
HDL-cholesterol	1,37 (0.77-2.11)	1,37 (0.74-2.44)	0,892

The results presented in Table 3 show an assessment of the risk of venous thrombosis in relation to the type of hyperlipoproteinemia, after adjustment for confounding factors, and clearly suggest a statistically significant effect of type IIa hyperlipoproteinemia on the risk of venous thrombosis, given the fact that fully adjusted OR was 1.99 (CI 1.01-3.90), indicating that this type of hyperlipoproteinemia doubles the risk for venous thrombosis.

**Table 3:** The risk of venous thrombosis in relation to the type of hyperlipoproteinemia * adjusted for gender and age, † Adjusted for gender, age, and obesity

Type of hyperlipoproteinemia	Patients	Controls	OR^*^	OR^†^
Without hyperlipoproteinemia	31 (31)	45 (45)	1,0 (Reference)	1,0 (Reference)
Type IIa	42 (42)	29 (29)	2,05 (1.05-4.00)	1,99 (1.01-3.90)
Type IIb	22 (22)	19 (19)	1,25 (0.84-1.85)	1,22 (0.79-1.84)
Type IV	5 (5)	6 (6)	0,99 (0.64-1.55)	0,89 (0.52-1.54)
Lp (a) hyperlipoproteinemia	20 (20)	12 (12)	1,81 (0.83-3.95)	1,85 (0.84-4.04)

## Discussion

Given the fact that thrombosis is a significant cause of mortality and morbidity in developed countries, scientists and medical professionals have devoted themselves to studying the etiology and pathophysiological mechanisms leading to it, which could potentially serve as a tool for adequate prevention and treatment. In previous years, the main focus was directed to arterial thrombosis and its most common complications, myocardial infarction, and ischemictype cerebrovascular stroke, but lately, the focus has been shifted to venous thrombosis. A number of different risk factors that have been proven to contribute to the occurrence of venous thrombosis are indicative of the multifactorial etiology in the onset of this disease and the relationship between arterial and venous thrombosis, and the risk factors common to these twothrombotic processes is of utmost importance. Research conducted by Prandoni P et al. [20] showed the relationship between atherosclerosis and the occurrence of venous thrombosis, based on which it was assumed that common etiological factors contribute to the onset of these diseases. Among them, the influence of hyperlipoproteinemia is of particular interest.

Our study included a total of 200 subjects, 100 of whom had experienced venous thrombosis and 100 laboratory and clinically proven healthy volunteers who had never experienced venous thrombosis. The results showed, above all, a higher percentage of hyperlipoproteinemia in the patient group than in the control group (69% vs. 45%), consistent with the results of the study by Vaýo A et al. [21] suggesting that hyperlipoproteinemia might be a risk factor for venous thrombosis. Additionally, a study conducted by Ray JG, et al. [22] showed that usage of hypolipemics, particularly statins, reduces the risk of venous thrombosis. The results of our study also indicate a more frequent occurrence of Lp (a) hyperlipoproteinemia in subjects in the patient group than in control subjects (20% vs. 12%). The results of the study by Nowak-Goottl et al. [23] showed higher levels of Lp (a) lipoprotein in the serum of patients with DVT. The significance of elevated serum Lp (a) lipoprotein was also demonstrated in the study conducted by Sofi et al. [24]. On the other hand, the research by McColl MD et al. [25] did not show a statistically significant elevation of serum Lp (a) in DVT patients. Comparison of mean values of individual lipid status parameters between the patient group and control group of healthy subjects showed statistically significant difference only when it came to LDLcholesterol values (3.84 mmol/L vs. 3.45 mmol/L p= 0.005). Similar results were obtained by Albert W et al. [26]. More over, a study by Kawasaki et al. [27] showed a significant impact of hypertriglyceridemia and hypercholesterolemia as risk factors for DVT. Deguchi et al. demonstrated the importance of LDLcholesterol as a risk factor for venous thrombosis, as well [28]. Hyper lipo pro teinemia type distribution showed a higher prevalence of type IIa hyperlipoproteinemia in the patient group compared to the control group (42% vs. 29%) as well as type IIb (22% vs. 19%), which is consistent with the results of Carvalho et al. [29]. Such results were also shown in the study of Deguchi et al. [28].

Unconditional logistic regression was used to assess the relative risk of venous thrombosis in patients with different types of hyperlipoproteinemia, and it was adjusted for age, gender, and obesity, as potential confounders. »Confounding« is a type of bias, implying a situation in which the effect of variables whose influence on a parameter we examine is mixed with the influence of another variable, which can cause bias, that is, a gross error in the interpretation of the obtained results. In other words, the »confounder« must have a proven relationship to the disease (as an immediate causative factor but not as an effect of the disease), as well as with the factor whose causal relationship to the disease is being examined. If the potential impact of »confounding« is not taken into account when designing the study, there is a risk of misinterpretation of the results. Therefore, in our study, we paid particular attention to eliminating the influence of »confounding« variables in statistical data processing and interpretation of the obtained results. Using unconditional logistic regression to evaluate the effect of different types of HLP on the risk of deep vein thrombosis, we have found that type IIa hyperlipoproteinemia is associated with a nearly doubled risk for deep vein thrombosis (OR 1.99; Cl 1.01-3.90), while type IIb, IV, or hyperLp (a) lipoproteinemia didn't influence this risk (OR 1.22; 95% Cl 0.79-1.84; OR 0.89; 95% Cl 0.52-1.54 OR 1.85; 95% CI 0.84-4.04). In light of our results, hypercholesterolemia can be identified as one of the potential risk factors for the development of DVT. By promoting endothelial dysfunction, it can influence complex regulation of the hemostatic mechanism, triggered by decreased concentrations of tissue factor pathway inhibitor (TFPl) and thrombomodulin. TFPI is synthesized mainly in endothelial cells, and it is detected that the free form of TFPI is transferred to the LDL/VLDL fraction due to the increase in LDL, which may cause a reduction in endothelial cell-associated TFPI. Increased concentrations of cholesterol can be treated with statins, and there is some evidence that these drugs decrease thrombosis risk, not only by lowering cholesterol levels but by stabilizing endothelial cells as well. As the majority of studies dealing with this topic have a small number of participants, a welldesigned meta-analysis could give us a final answer on whether we should consider hypercholesterolemia as a risk factor for venous thrombosis and use statins in the prevention of this disease.

## Conclusion

We conclude that type IIa hyperlipoproteinemia doubles venous thrombosis risk.

## Author declaration

Authors certify that the manuscript represents a valid piece of work and neither this manuscript nor one with substantially similar content under named authorship has been published or is being considered for publication elsewhere. The authors have participated in the research and the shaping of the manuscript.

## Conflict of interest statement

The authors have no conflicts of interest to declare. The authors give consent to the submission and publication of the work. Authors disclose no relationship to any organization or industrial manufacture in any material discussed.
